# Adaptation of the World Health Organization (WHO) Safe Surgery Checklist for Use With Cesarean Sections: Implementation and Outcomes With the Safe Cesarean Section Checklist

**DOI:** 10.7759/cureus.61330

**Published:** 2024-05-29

**Authors:** Vaishnavi J Patel, Peter G Napolitano, Eileen A Hemman, Peter E Nielsen, Shad Deering

**Affiliations:** 1 Office of Research and Innovation, University of the Incarnate Word School of Osteopathic Medicine, San Antonio, USA; 2 Department of Obstetrics and Gynecology, University of Washington, Seattle, USA; 3 Department of Obstetrics and Gynecology, Madigan Army Medical Center, Tacoma, USA; 4 Department of Obstetrics and Gynecology, University of Texas at San Antonio, San Antonio, USA; 5 Department of Obstetrics and Gynecology, Baylor College of Medicine, San Antonio, USA

**Keywords:** cesarean section complications, obstetrical surgery, surgical staff safety, who surgical safety checklist, surgical safety, obstetrics, c-section, patient safety, checklists, cesarean section

## Abstract

Introduction

The World Health Organization (WHO) Safe Surgery Checklist significantly decreases morbidity and mortality in regular operating room cases. However, significant differences in workflow and processes exist between regular operating room cases and cesarean sections performed on the labor and delivery unit. The aim of this study is to adapt the WHO Safe Surgery Checklist for the labor and delivery unit and cesarean sections to improve communication and patient safety.

Methods

A multidisciplinary team consisting of all major stakeholders reviewed and revised the WHO Safe Surgery Checklist making it more applicable to cesarean section operations. The new Safe Cesarean Section Checklist was tested and then integrated into the electronic medical record and utilized on the labor and delivery unit. A specific cesarean section safety attitudes questionnaire was developed, validated, and administered prior to and one year after implementation.

Results

Usage of the Safe Cesarean Section Checklist was greater than 95% after initial implementation. Significant improvements were reported by the staff on the cesarean section attitudes questionnaire for several key areas including the feeling that all necessary information was available at the beginning of the procedure, decreases in communication breakdowns and delays, and fewer issues related to not knowing who was in charge during the procedure.

Discussion

Implementation of the Safe Cesarean Section Checklist was successfully adopted by the staff, and improvements in staff perceptions of several key safety issues on our unit were demonstrated. Additional studies should be undertaken to determine if clinical outcomes from this intervention are comparable to those seen with the use of the WHO Safe Surgery Checklist.

## Introduction

The use of a preoperative checklist in the operating room is recommended by the World Health Organization (WHO) as this has been demonstrated to significantly decrease medical errors, including death [1-3]. In one report, just this simple tool resulted in a 36.6% decrease in mortality [1]. The preoperative checklist described in the WHO study included three distinct steps [2]: (1) preoperative brief (prior to the administration of anesthesia), (2) time-out (before skin incision), and (3) sign-out (before the patient leaves the operating room (OR)).

While this process has been integrated into the main operating rooms at many institutions, labor and delivery (L&D) units present a very different environment and pose unique challenges [4]. For example, L&D units include a combination of sometimes competing services which traditional operating rooms do not. These include an emergency department for pregnant patients, active laboring patients at term as well as preterm, and management of medically complex antepartum patients, in addition to the traditional operating room setting. While cesarean deliveries are often planned, many occur at any time during the day or night, pulling key personnel and resources from the rest of the unit and other patients [5]. In addition, the obstetric team must consider two or more patients and multiple services (obstetrics, pediatrics, and anesthesia) with every procedure [6]. These factors require careful consideration of the timing and composition of each team due to a dynamically changing environment on the unit and may require contingencies to cover other emergencies during the scheduled cesarean delivery. Based on the complexity of the L&D unit, communication and coordination are even more critical [7].

Given the key differences between the surgical and obstetrical environments, we felt it was necessary to modify the WHO Safe Surgery Checklist to fit the reality of the obstetric unit. While keeping the three defined steps of the validated checklist, we adapted them to include the unique issues and multiple specialties that must work together during a cesarean delivery.

## Materials and methods

A multidisciplinary team consisting of nurses, resident and staff physicians from anesthesia, pediatrics, and obstetrics and gynecology (OB/GYN), scrub technicians, and our patient safety nurse was assembled to create the Safe Cesarean Section Checklist. Based on evidence that the WHO Safe Surgery Checklist could improve outcomes in the operating room, the team decided to retain the same framework for the checklist, the timing for when the checklist would be used, and the general questions that would be asked. After a review of the specific components by our team, all items that were relevant to a cesarean section operation at our institution were included, which included nearly all of them [1]. Next, the current literature was reviewed, and obstetric-specific tasks were added [8]. A final review by the checklist team produced the initial version of the Safe Cesarean Section Checklist.

The initial version of the Safe Cesarean Section Checklist was then implemented for a two-week trial period in the L&D unit. During this time, a departmental policy detailing the program was drafted and signed by the department chief. This policy outlined both the reason for the checklist and the requirement that it be used.

At the end of the initial trial period, the checklist was revised based on feedback from the end users and then presented in its final version to the department staff at morning reports for involved departments and during nursing shift changes on multiple occasions in order to reach as many providers as possible prior to implementation. The new departmental policy was discussed during this time as well. The Safe Cesarean Checklist was also integrated as a stand-alone note in the electronic medical record, so that it could be accessed both at the nursing station for the preoperative briefing and in the operating room for the time-out and sign-out portions.

Our implementation process adhered to the Quality Improvement Cycle for Healthcare, reflecting a systematic and iterative approach to enhance the safety and effectiveness of cesarean section procedures [9]. This conceptual framework informed the development, trial, and refinement stages of the Safe Cesarean Section Checklist. By incorporating the principles of the Quality Improvement Cycle, we ensured ongoing assessment, feedback integration, and continuous improvement, aligning our methodology with established best practices in healthcare quality improvement [9].

**Figure 1:**
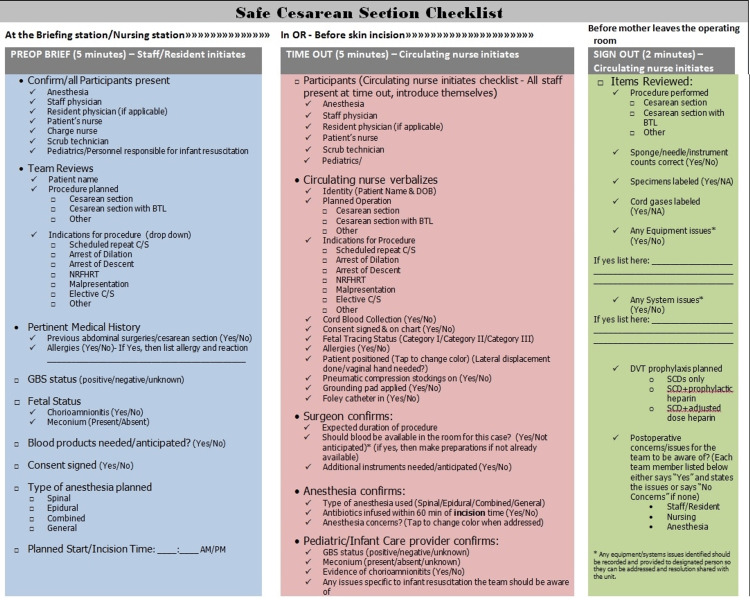
Safe Cesarean Section Checklist PREOP: preoperative; BTL: bilateral tubal ligation; C/S: cesarean section; GBS: group B *Streptococcus*; DOB: date of birth; DVT: deep vein thrombosis; SCD: sequential compression device

**Figure 2:**
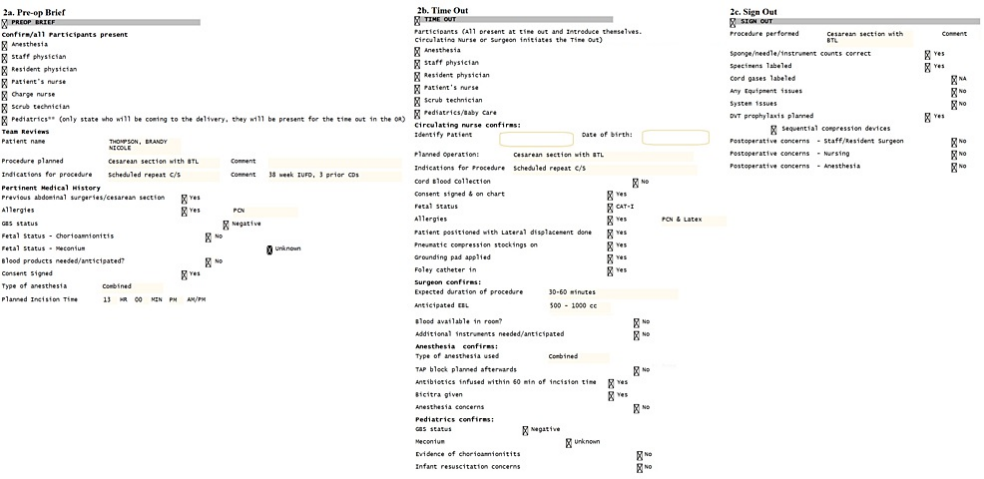
Screenshots of the Safe Cesarean Checklist in electronic medical record Figure 2a indicates the preoperative brief portion of the electronic medical record. Figure 2b is the time-out portion. Figure 2c is the sign-out portion. The names and patient information in this image have been altered in compliance with HIPAA. OR: operating room; BTL: bilateral tubal ligation; C/S: cesarean section; IUFD: intrauterine fetal demise; CD: cesarean delivery; PCN: penicillin; GBS: group B *Streptococcus*; EBL: estimated blood loss; TAP: trans-abdominal plane; DVT: deep vein thrombosis; HIPAA: Health Insurance Portability and Accountability Act

**Figure 3:**
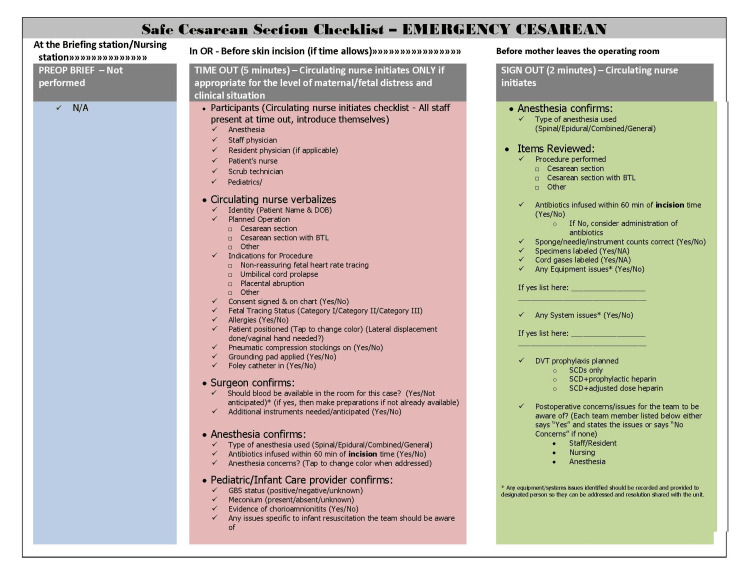
Emergent Safe Cesarean Section Checklist PREOP: preoperative; BTL: bilateral tubal ligation; C/S: cesarean section; GBS: group B *Streptococcus*; DOB: date of birth; DVT: deep vein thrombosis; SCD: sequential compression device

The final components of the Safe Cesarean Section Checklist can be seen in Figure 1, and an example of how it appears in the electronic medical record is shown in Figure 2. It was explained to all members of the team that the Safe Cesarean Section Checklist was to be used for all scheduled and indicated cesarean sections. In the case of a truly emergent cesarean section, the preoperative brief was not performed, and only as much of the time-out was done as possible based on the staff physician's decision about the fetal/maternal status. At the end of the procedure, the sign-out portion of the checklist was still to be used. After feedback from members of the unit, we modified the standard checklist for these emergency procedures (Figure 3).

After the program was implemented, a patient safety representative within the department was assigned to review the electronic charts for all patients who had a cesarean section. If there were charts where the checklist should have been used, and it was not, this information was provided to the head nurse of the L&D unit, and the chief of the obstetrics service and the staff involved were counseled regarding the need for complete documentation and compliance with departmental policy.

**Figure 4:**
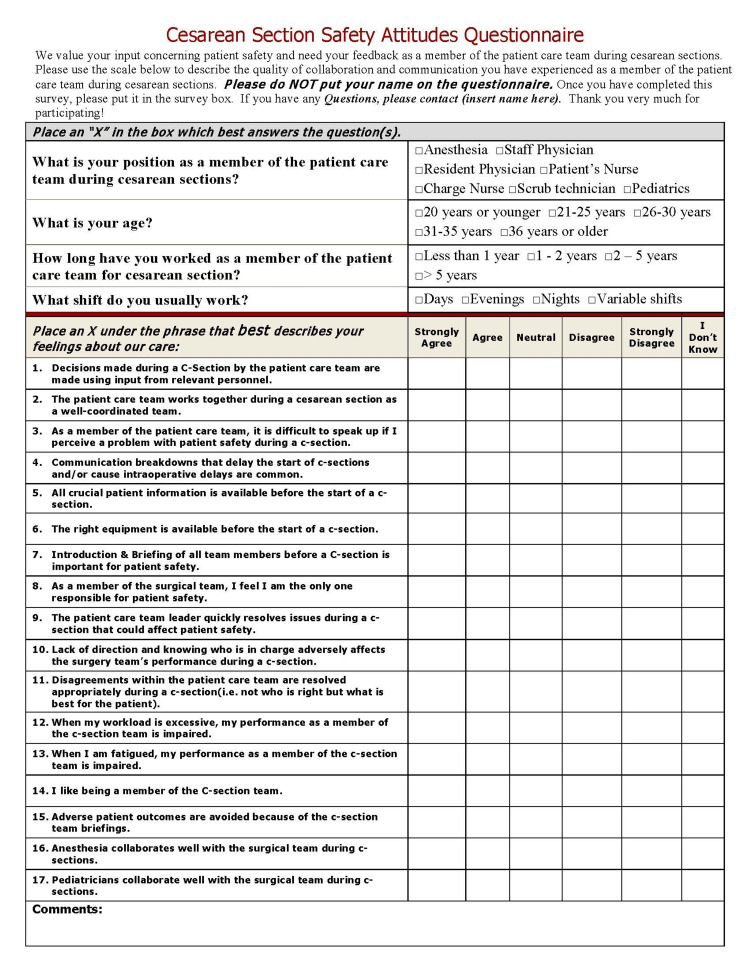
Cesarean section safety attitudes questionnaire

To determine if there was an impact related to the checklist's implementation, a staff patient safety attitude survey specific to cesarean sections was developed. One of the important steps prior to employing a user-developed instrument is to determine its validity. Prior to the development of the checklist, a review of the literature was conducted to establish the dimensions of patient safety and a basic set of elements for clinical checklists currently being used. Items were generated or tailored to address each dimension, assembled into a survey format, and arranged in a suitable sequence. Subject matter experts in cesarean sections were given the initial survey to provide feedback on the questions. The last step prior to using the survey was to reformat the questions into a Content Validity Index (CVI) template [10]. Six subject matter experts rated each question on the relevance to a dimension of patient safety and commented and identified any missing dimension(s) or gaps in the survey. A Likert scale of 1-4 was used for the rating scale (1=not relevant; 4=very relevant). Using Lynn's recommendations, five out of six raters had to endorse each item as a 3 or 4 to establish content validity beyond the 0.05 level of significance [10]. All questions had a CVI of 1.0 with an overall CVI of 0.99 (99% agreement). Several minor wording changes were implemented based on the experts' feedback. Demographic questions and unit characteristics were added to the survey in the final form. This new questionnaire was then administered to the L&D staff prior to the implementation of the Safe Cesarean Section Checklist and then again one year later. In the pre- and post-implementation periods, these were handed out by the patient safety nurse at both the physician's morning reports and nursing checkout, and for the post-implementation period, copies of the survey were also placed in the staff mailboxes. The questionnaire itself can be seen in Figure 4.

In order to evaluate the survey responses, we chose to analyze the results in an aggregate manner, looking at the change in attitudes towards the potential changes anticipated (e.g., differences in the number of agree/strongly agree or disagree/strongly disagree in the pre- and post-implementation time periods). For this, we utilized non-parametric statistics and chose a p-value of 0.05 as significant. This intervention was reviewed by the Department of Clinical Investigation and deemed to be consistent with a quality improvement project and exempt status.

## Results

**Figure 5:**
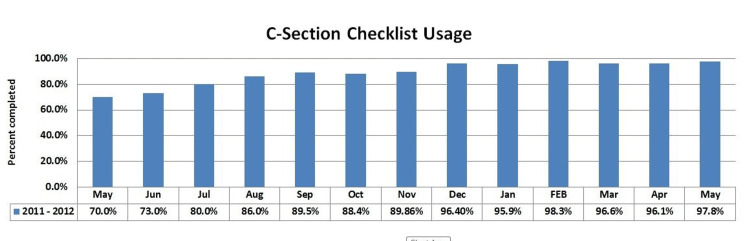
Safe Cesarean Section Checklist utilization

A total of 74 surveys were distributed during the pre-implementation period with 61 returned for an 82% response rate and 97 in the post-implementation period with 58 returned for a 60% response rate. The distribution between physicians and nursing personnel that responded each time was not significantly different (43% physicians/57% nurses before vs. 39% physician/61% nurses afterwards; p=0.7). The usage rate of the Safe Cesarean Section Checklist increased rapidly over the first several months of the program, and this is demonstrated in Figure 5, with over 95% consistent usage after six months.

While there were no differences in the pre- and post-implementation responses for most of the questions, we did identify three specific areas where statistically significant improvements were reported. The first was that the percentage of staff that felt all critical information was available prior to beginning the cesarean section (agreed/strongly agreed) increased from 82% to 97% (p=0.011). The second was significantly fewer staff reported feeling that a lack of direction and knowing who was in charge adversely affected the surgery team's performance during a cesarean section 65% vs. 46.5% (p=0.034). The third was that respondents felt that breakdowns in communication and delays were less common with only 48% saying they disagreed/strongly disagreed with the statement "Communication breakdowns that delay the start of cesarean sections and/or cause intraoperative delays are common" prior to the checklist vs. 67% afterwards (p=0.045). The other questions asked did not show significant differences (Table 1 and Table 2).

**Table 1:** Cesarean section safety attitudes questionnaire: agree/strongly agree pre- and post-implementation of the Safe Cesarean Section Checklist P-values were calculated using the chi-squared analysis. The sample size (n) is based on the number of participants who completed each questionnaire item. Within the parenthesis is the percentage of those who answered the questionnaire item with agree or strongly agree using the sample size for that specific questionnaire item. Pre-implementation indicates the responses before the Safe Cesarean Section Checklist was implemented, while post-implementation indicates the responses after six months of over 95% usage of the Safe Cesarean Section Checklist

Questionnaire item	Pre-implementation agree/strongly agree (n=58-61)	Post-implementation agree/strongly agree (n=27-55)	P-value
Decisions are made with input from relevant personnel	57 (93.4%)	55 (94.8%)	0.748
The cesarean section team is a well-coordinated team	58 (95.1%)	57 (98.3%)	0.334
All crucial patient information is available before the start of the cesarean section	50 (82.0%)	56 (96.6%)	0.011
The right equipment is available before the start of the cesarean section	58 (98.3%)	56 (98.2%)	0.980
Introduction and briefing of all is important for patient safety	56 (91.8%)	49 (87.5%)	0.443
The patient care team leader quickly resolves issues during cesarean section	49 (84.5%)	43 (74.1%)	0.264
Lack of direction regarding who is in charge adversely affects performance	40 (65.2%)	27 (46.5%)	0.034
Disagreements are resolved appropriately	50 (84.7%)	43 (76.8%)	0.267
Excessive workload impairs my performance	33 (55.9%)	40 (69.0%)	0.146
Fatigue impairs my performance	39 (63.9%)	40 (69.0%)	0.561
I like being a member of the cesarean section team	55 (90.2%)	52 (89.7%)	0.927
Adverse patient outcomes are avoided by cesarean section briefs	42 (72.4%)	43 (74.1%)	0.834
Anesthesia collaborates well	51 (86.4%)	49 (84.5%)	0.764
Pediatrics collaborates well	41 (67.2%)	31 (54.4%)	0.153

**Table 2:** Cesarean section safety attitudes questionnaire: disagree/strongly disagree pre- and post-implementation of the Safe Cesarean Section Checklist P-values were calculated using the chi-squared analysis. The sample size (n) is based on the number of participants who completed each questionnaire item. Within the parenthesis is the percentage of those who answered the questionnaire item with disagree or strongly disagree using the sample size for that specific questionnaire item. Pre-implementation indicates the responses before the Safe Cesarean Section Checklist was implemented, while post-implementation indicates the responses after six months of over 95% usage of the Safe Cesarean Section Checklist

Questionnaire item	Pre-implementation disagree/strongly disagree (n=60-61)	Post-implementation disagree/strongly disagree (n=57-58)	P-value
It is difficult to speak up if I perceive a problem	55 (90.2%)	52 (89.7%)	0.700
Communication breakdowns and delays are common	29 (48.3%)	38 (66.7%)	0.045
I feel I am the only one responsible for patient safety	55 (91.7%)	53 (91.4%)	0.955

## Discussion

Preventing surgical morbidity is a key component of improving patient safety in any specialty. For the main operating room, the implementation of a simple checklist process has been shown to significantly improve outcomes with decreases in both operative morbidity and mortality [1]. Other specialties have followed suit and created their own specialty checklists, such as the American Academy of Ophthalmology [11]. This concept and potential for improvement directly applies to obstetrics as over 1.2 million cesarean sections are performed annually in the United States with 32.1% of all births being by cesarean section [12]. There are, however, several key differences in the obstetric operating room, where two patients must be considered with every procedure and multiple departments are required to communicate on both scheduled and non-scheduled cases at all hours of the day and night [4-7].

While we did not see improvement in every area addressed in the cesarean section attitudes survey, we did find several important positive changes. These appear to be related to improved communication leading to less delay in starting procedures, ensuring that all relevant information was available at the beginning of the procedure, and clarifying who was in charge to help things move ahead in a timely manner. These are all in line with expected changes when communication strategies such as checklists are implemented [13].

Limitations of our study include the fact that it was implemented at a single institution as a quality improvement where TeamSTEPPS training had already been in place for several years prior to introducing the checklist and the dynamic nature of the L&D unit and the inability to account for other factors influencing the survey results over the 12-month time period examined [14]. We feel that the prior teamwork and communication training contributed to the high positive baseline responses we received on the pre-implementation survey, though if that is the case, then it makes the changes noted even more significant. We also acknowledge that we are reporting changes in staff attitudes rather than objective outcomes, though we feel that this initial work helps to provide a foundation to begin to look at these as well. With regard to undertaking the implementation of a checklist tool for quality improvement, we feel that there are several important lessons to be learned from our experience.

First, to implement even a simple intervention such as this checklist, planning and inclusion of the entire patient care team are required [15]. This was done at our institution by including key stakeholders, such as the head nurse of the unit, representatives from the different departments that participate in a cesarean section (pediatrics, obstetrics, and anesthesia), surgical technicians/assistants, and the patient safety manager, in the final decisions on how best to perform the preoperative brief (timing and location) as well as getting their feedback on any additional items that needed to be included in order to cover local institutional issues or removed in order to make the checklist easier to use. By having the team involved in the creation and implementation process, we feel we obtained much more buy-in than if the program had simply been created in a vacuum and then presented as a requirement. After this, monitoring usage is a critical step after the initial implementation. As the checklist requires a change in workflow, even when the appropriate staff is involved in the integration process, it will still take practice to make it run smoothly.

Part of the reason that the WHO Safe Surgery Checklist was successful is that the researchers allowed each institution to determine how exactly to fit the checklist into their workflow [16]. While the sections remained the same (preoperative brief/time-out/sign-out), the hospitals were allowed to use the same principles but tailor it for their workflow processes. This is a key component to making the checklist work at any institution. At our hospital, we conducted our preoperative brief immediately after our morning checkout rounds and discussed any scheduled cesarean section cases for the day. For any other cesarean deliveries that became necessary during the day or night, we would gather the team together and conduct our preoperative brief when the decision was made. The sign-out and time-out sections were always done in the operating room.

Other keys we identified in the implementation process included the following: (1) Practice with the checklist and see how it fits into the workflow of your unit and then make changes based on the staff's recommendations. Gather feedback on the items on the checklist and determine if there are any elements missing that may be unique to your institution. (2) Write a departmental policy/protocol explaining how the Safe Cesarean Section Checklist will be implemented and the requirement/explanation for doing so. (3) Introduce the checklist and brief all departments that will be involved (anesthesia, pediatrics, obstetrics, nursing, etc.). We did this by developing PowerPoint briefings at morning reports and demonstrating to the clinical teams at change of shift to ensure standardized education was given. (4) Assign a specific person to be responsible for monitoring usage/compliance with the checklist. This person should be specifically named in the departmental policy/protocol. We recommend that this should be reviewed on at least a weekly basis at the beginning of implementation. To make this happen, it is critical to assign specific personnel to track usage/compliance with the checklist and to ensure that follow-up for any issues identified is accomplished. At our institution, a specific staff is identified to monitor daily usage of the checklist. If it is not utilized in a non-emergent case, the head nurse is notified who then follows up with the individual nurse assigned to the case. This increased our usage, and we saw a dramatic improvement in completion rates to greater than 98%.

While we chose to integrate the checklist into our electronic record, we recognize that not all institutions have electronic records or the ability to modify them even if they do. In these cases, the checklist may be done on paper [17]. Exactly how this occurs will depend on the institution's analysis of how it best fits into their workflow.

One important thing to note about the Safe Cesarean Checklist is that it is not meant for emergency cesarean sections. We do not advocate delaying delivery for the checklist in truly emergent situations. However, after the operation, there is a sign-out that is specific for emergency cesarean sections, and this should be used in those cases. Based on feedback from the team, we modified the original checklist and created a version to be used for truly emergent cesarean sections (Figure 3).

Clearly identifying a person to follow up on issues identified by the checklist is also critical so that any systems or equipment issues can be immediately corrected and the team notified of the findings [15]. This step increases both buy-in and the credibility of the program and demonstrates policy in action to improve patient safety. Not taking these steps risks making the checklist appear like just another requirement that is not followed up and does not appear to make a difference.

## Conclusions

The WHO Safe Surgery Checklist has been shown to significantly decrease morbidity and mortality. However, as discussed, the significant differences between scheduled operating room cases and cesarean sections performed on the L&D unit required adaptation at our institution. In an attempt to realize the same benefits seen with the WHO Safe Surgery Checklist on the L&D unit, we modified and implemented a Safe Cesarean Section Checklist at our institution and found that it improved the team's perception of safety in several areas. This Safe Cesarean Section Checklist brings evidence-based safety practices into the L&D operating room and can provide real-time data that can be used to improve patient safety.

Though we feel that the implementation of the Safe Cesarean Section Checklist is an important step forward in patient safety, usage should be monitored and feedback on lessons learned provided to the staff. Only then will the organization realize all of the potential benefits of the Safe Cesarean Section Checklist. While we have demonstrated subjective improvement in several areas with the use of the checklist, additional study is needed in order to determine if these will translate to objective improvement in patient outcomes similar to those notes in the original WHO Surgical Safety Checklist study.

## References

[1] Ramsay G, Haynes AB, Lipsitz SR, Solsky I, Leitch J, Gawande AA, Kumar M (2019). Reducing surgical mortality in Scotland by use of the WHO Surgical Safety Checklist. Br J Surg.

[2] (2024). WHO guidelines for safe surgery: Safe surgery saves lives. https://www.who.int/publications-detail-redirect/9789241598552.

[3] Chhabra A, Singh A, Kuka PS, Kaur H, Kuka AS, Chahal H (2019). Role of perioperative surgical safety checklist in reducing morbidity and mortality among patients: an observational study. Niger J Surg.

[4] Plough A, Henrich N, Galvin G, Shah NT (2018). Common challenges managing bed and staff availability on labor and delivery units in the United States: a qualitative analysis. Birth.

[5] Torloni MR, Betran AP, Souza JP, Widmer M, Allen T, Gulmezoglu M, Merialdi M (2011). Classifications for cesarean section: a systematic review. PLoS One.

[6] Iravani M, Zarean E, Janghorbani M, Bahrami M (2015). Women's needs and expectations during normal labor and delivery. J Educ Health Promot.

[7] Maxfield DG, Lyndon A, Kennedy HP, O'Keeffe DF, Zlatnik MG (2013). Confronting safety gaps across labor and delivery teams. Am J Obstet Gynecol.

[8] Duff P (2010). A simple checklist for preventing major complications associated with cesarean delivery. Obstet Gynecol.

[9] Varkey P, Reller MK, Resar RK (2007). Basics of quality improvement in health care. Mayo Clin Proc.

[10] Lynn MR (1986). Determination and quantification of content validity. Nurs Res.

[11] (2023). Surgical safety checklist. https://www.aao.org/asset.axd?id=ba210a8b-68ac-4911-b537-f5d8ae53c16a.

[12] (2023). Births - Method of delivery. https://www.cdc.gov/nchs/fastats/delivery.htm.

[13] Russ S, Rout S, Sevdalis N, Moorthy K, Darzi A, Vincent C (2013). Do safety checklists improve teamwork and communication in the operating room? A systematic review. Ann Surg.

[14] (2024). Agency for Healthcare Research and Quality. http://teamstepps.ahrq.gov/abouttoolsmaterials.htm.

[15] Levy SM, Senter CE, Hawkins RB (2012). Implementing a surgical checklist: more than checking a box. Surgery.

[16] Saturno PJ, Soria-Aledo V, Da Silva Gama ZA, Lorca-Parra F, Grau-Polan M (2014). Understanding WHO surgical checklist implementation: tricks and pitfalls. An observational study. World J Surg.

[17] Kulp L, Sarcevic A, Cheng M, Zheng Y, Burd RS (2019). Comparing the effects of paper and digital checklists on team performance in time-critical work. Proc SIGCHI Conf Hum Factor Comput Syst.

